# 原发免疫性血小板减少症诊疗现状——多中心回顾性临床研究

**DOI:** 10.3760/cma.j.cn121090-20241104-00431

**Published:** 2025-06

**Authors:** 求哲 魏, 沁颖 谢, 琳琳 黄, 国林 袁, 惠丽 蔡, 道滋 姜, 元艳 唐, 世民 陈, 宏波 任, 恒 梅

**Affiliations:** 1 华中科技大学同济医学院附属协和医院血液科，武汉 430022 Department of Hematology, Union Hospital, Tongji Medical College, Huazhong University of Science and Technology, Wuhan 430022, China; 2 襄阳市中心医院血液科，襄阳 441021 Department of Hematology, Xiangyang Central Hospital, Xiangyang 441021, China; 3 宜昌市中心人民医院血液科，宜昌 443003 Department of Hematology, Yichang Central People's Hospital, Yichang 443003, China; 4 武汉大学人民医院血液科，武汉 430060 Department of Hematology, Renmin Hospital of Wuhan University, Wuhan 430060, China; 5 荆州市中心医院血液科，荆州 434000 Department of Hematology, Jingzhou Central Hospital, Jingzhou 434000, China; 6 黄石市中心医院血液科，黄石 435000 Department of Hematology, Huangshi Central Hospital, Huangshi 435000, China; 7 荆州市第一人民医院血液科，荆州 434000 Department of Hematology, Jingzhou First Peoples's Hospital, Jingzhou 434000, China

**Keywords:** 血小板减少症, 回顾性分析, 生活质量, Thrombocytopenia, Retrospective analysis, Quality of life

## Abstract

**目的:**

探究湖北省部分地区原发免疫性血小板减少症（ITP）患者的诊疗情况及生活质量。

**方法:**

回顾性分析2020年1月至2022年12月期间湖北省七家医疗中心ITP患者的年龄、病程、症状、诊断及治疗情况，通过ITP患者评估问卷（ITP-PAQ）线上调查随访患者的生活质量情况。

**结果:**

1033例患者纳入研究，新诊断、持续性、慢性ITP患者占比分别为39.8％（411例）、19.1％（197例）、41.1％（425例）。76.1％（786例）的患者有不同程度出血表现。糖皮质激素（600例，8.1％）和促血小板生成药物（430例，41.6％）是ITP患者首诊时选择最多的治疗药物，不良反应主要有腹泻（96例，9.3％）、肝功能不良（68例，6.6％）及血栓形成（52例，5.0％）。125例患者的ITP-PAQ调查显示，ITP患者在疲劳、睡眠、恐惧心理、运动、工作、社交多个方面得分均明显降低。

**结论:**

ITP疾病慢性化比例较高，糖皮质激素及促血小板生成药物是主要治疗药物。ITP患者的生活质量明显降低。

## Abstract (en)

**Objective:**

To review the diagnosis, treatment and quality of life of patients with primary immune thrombocytopenia（ITP）in seven medical centers in some areas of Hubei Province.

**Methods:**

A retrospective analysis was conducted on age, disease course, symptoms, diagnosis, and treatment status（including testing items, drug selection, and adverse reactions）of patients with ITP in seven medical centers in Hubei Province from January 2020 to December 2022. An online survey was conducted on the quality of life of patients using the ITP Patient Assessment Questionnaire（ITP-PAQ）.

**Results:**

Among the 1033 patients, those with newly diagnosed, persistent, and chronic ITP accounted for 39.8％, 19.1％, and 41.1％, respectively. Most patients exhibit varying degrees of bleeding. Regarding treatment, corticosteroids and thrombopoietin drugs are the most commonly chosen treatment drugs for ITP, and the adverse reactions to treatment mainly include diarrhea, liver dysfunction, and thrombosis. The ITP-PAQ survey of 125 patients revealed that ITP significantly impairs their life quality. Patients with ITP scored significantly lower in fatigue, sleep, fear, exercise, work, and social aspects.

**Conclusion:**

A relatively high proportion of patients with ITP progressed to the chronic phase. Corticosteroids and thrombopoietin drugs are the two main treatment drugs for ITP patients. The quality of life of patients with ITP is significantly reduced in multiple dimensions.

原发免疫性血小板减少症（ITP）是血液科常见的一种获得性、免疫性、出血性疾病。ITP占出血性疾病的1/3，国外报道的成人ITP年发病率为3/10万，儿童ITP年发病率为（2.2～5.3）/10万[1]。本研究对2020年1月至2022年12月期间湖北省七家医疗中心血液科收治的ITP患者的临床资料进行回顾性分析，旨在提高对ITP患者的临床诊治特点的认识，为今后ITP患者的诊断与治疗提供参考。

## 病例与方法

一、研究对象

收集2020年1月至2022年12月湖北省七家医疗中心（荆州市第一人民医院、武汉协和医院、荆州市中心医院、襄阳市中心医院、宜昌市中心人民医院、武汉大学人民医院、黄石市中心医院）收治的共1033例ITP患者的临床资料，研究方案经华中科技大学同济医学院附属协和医院伦理委员会批准（伦理批件号：UHCT-IEC-SOP-016-03-01）。

回顾性分析ITP患者的年龄、病程、症状、诊断及治疗情况（检测项目、药物选择、不良反应）。

对随访患者发放ITP患者评论问卷（ITP-PAQ）进行线上调查，共回收有效问卷125份，回收率12.1％。

二、诊断及排除标准

成人和儿童的ITP诊断标准参照《成人原发免疫性血小板减少症诊断与治疗中国指南（2020年版）》[2]和《中国儿童原发性免疫性血小板减少症诊断与治疗改编指南（2021版）》[3]。排除继发性血小板减少症。

三、患者生活质量评估

ITP-PAQ是专门针对ITP患者生活质量的调查工具，由Mathias等在2007年开发和验证，由10个维度、44个问题组成，分别为症状、乏力 / 睡眠、困扰、运动、心理、恐惧、生活质量、社会活动、女性生殖系统健康和工作[4]–[5]。每一个维度的所有条目都从初始评分转换为0～100的得分，0分表示最差的健康生活质量，100则代表最高的得分，患者的生活质量与得分呈正相关关系。

四、随访

通过查阅病历和电话进行随访，随访截止日期为2023年12月20日。

五、统计学处理

统计分析采用*R* 4.4.0软件进行。连续变量使用“例数”、“均数（均值±标准差）”描述，分类变量的描述使用“例（％）”描述。多组间连续变量采用单因素方差分析，无序分类变量采用Pearson卡方检验。双侧*P*<0.05表示差异有统计学意义。

## 结果

一、一般情况

共收集2020年至2023年在湖北省七家医疗中心确诊的ITP患者1033例。<18岁组115例（11.1％），18～60岁组596例（57.7％），>65岁组322例（31.2％）；新诊断ITP（病程小于3个月）411例（39.8％），持续性ITP（病程3～12个月）197例（19.1％）。慢性ITP（病程大于12个月）425例（41.1％）。928例患者（89.8％）来自湖北省内，105例（10.2％）患者来自其他省市。

二、出血情况

所有1033例患者中，247例（23.9％）无出血表现，440例（42.6％）有瘀点、瘀斑、血肿等皮下出血表现，308例（29.8％）有鼻出血、齿龈出血、口腔血疱、结膜出血等黏膜出血表现，38例（3.7％）有内脏或中枢神经系统等深部器官出血（表1）。

**表1:** 1 033例来自湖北省7家医疗中心的原发免疫性血小板减少症（ITP）患者诊疗情况［例（％）］

指标	结果
出血情况	
无出血	247（23.9）
皮下出血	440（42.6）
黏膜出血	308（29.8）
深部器官出血	38（3.7）
首诊治疗药物	
糖皮质激素	600（58.1）
静脉注射免疫球蛋白	188（18.2）
促血小板生成药物	430（41.6）
抗CD20单抗	63（6.1）
其他	94（9.1）
二线治疗药物	
促血小板生成药物	573（55.5）
抗CD20单抗	124（12.0）
其他	150（14.5）
治疗相关不良反应	
腹泻	96（9.3）
肝功能不良	68（6.6）
血栓	52（5.0）
其他	105（2.0）
复发（850例）	
有	391（46.0）
无	459（54.0）

三、治疗药物选择

ITP的治疗药物主要包括糖皮质激素、静脉注射免疫球蛋白、促血小板生成药物、抗CD20单抗及免疫抑制剂（硫唑嘌呤、环孢素A等）。首次就诊使用的治疗药物依次为糖皮质激素（600例，58.1％）、促血小板生成药物（430例，41.6％）、静脉注射免疫球蛋白（188例，18.2％）、其他药物（硫唑嘌呤、环孢素A、达那唑等）（94例，9.1％）、抗CD20单抗（63例，6.1％）。另外，410例（39.7％）患者接受联合用药治疗。二线治疗依次为促血小板生成药物（573例，55.5％）、其他药物（硫唑嘌呤、环孢素A、达那唑等）（150例，14.5％）、抗CD20单抗（124例，12.0％）。

四、治疗相关不良反应及复发情况

ITP患者治疗过程出现的不良反应有血栓（52例，5.0％）、肝功能不良（68例，6.6％）、腹泻（96例，9.3％）、其他（105例，10.2％）。在出现血栓事件的患者中，32例为动脉血栓，20例为静脉血栓，动脉血栓发生率高于静脉血栓（61.5％对38.5％）。血栓患者与未发生血栓患者的血小板生成素受体激动剂（TPO-RA）使用率分别为71.15％、57.29％（*P*＝0.048，*χ*^2^＝3.90），动脉、静脉血栓患者的TPO-RA使用率分别为75.0％（24例）、65.0％（13例）（*P*>0.05，*χ*^2^＝0.60）。随访获得850例患者的停药后复发情况，复发率为46.0％（391/850）。

五、患者生活质量调查

对随访ITP患者采用ITP-PAQ进行生活质量问卷调查，共计回收有效问卷125份。其中男性54例（43.2％），女性71例（56.8％）。将问卷结果转换为0～100分制，0分表示最差的生活质量，100分代表最好的生活质量，每个维度的分数取该维度所有条目分数的平均值。转换后125例患者在九个维度（未纳入女性生殖系统健康维度）的得分如表2所示。ITP患者的ITP-PAQ平均得分为58.86分，其中生活质量维度的分数最低（42.67分），困扰（54.80分）、运动（55.10分）、心理（56.10分）、恐惧（59.76）几个维度的得分均较低，可见ITP对患者的生活质量有明显影响。按照年龄分组，18～65岁组的生活质量平均得分略低于<18岁及>65岁组，但差异均无统计学意义（*F*＝2.17，*P*>0.05）（表3）。在性别方面，男性组在各个维度的平均得分均略高于女性组，但差异均无统计学意义（*t*＝1.19，*P*>0.05）（表2）。所有接受调查的患者中，有11例（8.8％）患者认为ITP对其生活质量影响极大，39例（31.2％）患者认为影响很大或影响较大，仅11例（8.8％）患者认为ITP对其生活质量没有影响。疲劳乏力及睡眠质量差是ITP患者常见的症状，在本调查中，50例（40.0％）患者报告其总是/频繁/经常入睡困难，64例（51.2％）患者报告其总是/频繁/经常夜间觉醒。另有63例（50.4％）患者感到总是/频繁/经常身体疲乏。在运动情况、恐惧心理、社会活动、工作等方面，ITP患者亦受到不同程度的影响：112例（89.6％）患者认为ITP影响了自己的运动能力，以上结果提示医生在接诊随访ITP患者时，除了采用血小板计数、骨髓检查等实验室指标进行疗效评判，还应重视其疲劳症状、睡眠质量的改善并关注患者的情绪及心理状态。

**表2:** 125例原发免疫性血小板减少症（ITP）患者的ITP-PAQ各维度得分情况（*x*±*s*）

维度	总体（125例）	男性（54例）	女性（71例）
症状	72.10±14.92	73.23±15.37	71.24±14.62
乏力/睡眠	61.60±21.56	66.55±21.31	57.83±21.12
困扰	54.80±23.55	56.45±23.58	53.54±23.61
运动	55.10±27.86	56.25±30.79	54.23±25.61
心理	56.10±26.96	60.53±27.84	52.73±25.96
恐惧	59.76±21.60	61.30±23.05	58.59±20.51
生活质量	42.67±25.53	48.27±27.58	38.40±23.15
社会活动	63.25±23.34	63.77±25.56	62.85±21.67
工作	64.40±27.80	66.90±25.60	62.50±29.39
平均得分	58.86±18.29	61.47±20.76	56.88±16.04

**表3:** 各年龄组原发免疫性血小板减少症（ITP）患者的ITP-PAQ问卷得分情况

年龄组	例数	得分（*x*±*s*）
<18岁	9	65.84±24.81
18～45岁	46	58.11±18.70
>45～65岁	49	55.80±16.05
>65岁	21	66.08±17.02

## 讨论

以往研究显示，多达三分之二的成人ITP患者及20％～25％的儿童ITP患者可发生疾病的慢性化[6]。在本研究中，慢性和持续性患者占60.2％（622例）。本研究中ITP的一线治疗包括糖皮质激素及静脉注射免疫球蛋白。糖皮质激素是ITP患者的基本治疗药物之一，但较多的不良反应（如体重增加、疲乏、失眠、脱发、情绪症状等）限制了其长期使用[7]。对于糖皮质激素治疗无效或病情反复的ITP患者可应用二线治疗药物（促血小板生成药物、利妥昔单抗、达那唑、长春新碱、硫唑嘌呤等）。TPO是ITP患者主要的二线治疗药物，主要包括重组人血小板生成素（rhTPO）及TPO-RA两类。艾曲泊帕是目前最常使用的一种TPO-RA，在一项接受长期艾曲泊帕治疗（EXTEND）的大型临床研究中，85.8％的慢性ITP患者获得缓解，52％的患者获得25周及以上的持续缓解[8]，同时其在中国儿童慢性ITP治疗中也显示出良好的疗效及耐受性[9]。其他TPO-RA药物包括阿伐曲泊帕、海曲泊帕、芦曲泊帕、罗普司亭等。阿伐曲泊帕是一种新型的口服TPO-RA，与其他TPO-RA相比具有无肝毒性、无食物-药物相互作用的特点，有望成为慢性ITP患者二线治疗的新选择，并且在既往对艾曲泊帕、罗普司亭等TPO-RA反应不佳的患者中也具有高反应率[10]。海曲泊帕是中国自主研发的新一代小分子TPO-RA，已于2021年被国家食品药品监督管理局批准用于治疗既往对糖皮质激素、静脉注射免疫球蛋白等治疗反应不佳的成人慢性ITP患者。以上两种药物均已在中国完成了Ⅲ期临床试验，在成人慢性ITP患者中显示了良好的安全性、有效性及耐受性[11]–[12]。此外，在已完成并发表的临床试验中，艾曲泊帕、罗米司亭在成人和儿童ITP患者中显示出生活质量的明显改善[13]–[18]。

在治疗方式方面，一项Meta分析显示，与单药治疗相比，联合治疗方案具有更好的早期反应和持续反应[19]。本研究中，选择联合治疗的患者占总随访患者的39.7％，对停药时间的统计显示大部分联合治疗患者（85.7％）在一年内停药。目前，已在ITP患者中使用过的联合方案有不同作用机制的药物联合（如TPO-RA联合糖皮质激素）、不同治疗时间窗的药物联合（如利妥昔单抗联合rhTPO）及不同机制和不同时间窗的药物联合（如利妥昔单抗联合糖皮质激素）等。然而，目前仍没有基于临床循证医学的统一ITP治疗方案及具体药物联合方案，且仍缺少大规模的临床研究数据对不同联合治疗方案的疗效进行比较[20]。

ITP患者的治疗不良反应与复发一直是临床医生重点关注的问题。本研究中，ITP患者出现的治疗不良反应按出现频率排序依次为腹泻（96例，9.3％）、肝功能不良（68例，6.6％）及血栓（52例，5.0％）。ITP患者的肝功能不良可能与促血小板生成药物艾曲泊帕相关。肝功能损伤是艾曲泊帕较严重的不良反应之一，据文献报道，高达10％的服用艾曲波帕的患者可能发生转氨酶升高，长期使用约4％的患者会出现高胆红素血症，该不良反应在一定程度上影响了其长期应用的安全性[21]。因此，使用艾曲泊帕治疗的ITP患者需要密切监测肝功能。另外，ITP患者发生动静脉血栓的风险均高于一般人群，且动脉血栓发生率（1.12％）高于静脉血栓发生率（0.22％）[22]。ITP患者血栓形成风险升高的原因目前仍不清楚，有研究者推测可能与血小板过度激活、抗磷脂抗体及巨核细胞的作用相关[23]。TPO-RA治疗与血栓栓塞事件的联系已在大量文献中进行过讨论，然而其是否明确增加ITP患者的血栓形成风险仍然存在争议。在两种TPO-RA药物（罗普司亭、艾曲泊帕）的单臂、长期TPO-RA试验中，6％的成年ITP患者报告了血栓栓塞事件[8],[14]。然而最近的一项包括25项研究的Meta分析显示，接受TPO-RA治疗的ITP患者与未使用TPO-RA的ITP患者相比血栓形成风险并未显著上升[24]。在本研究中，动脉血栓发生率高于静脉血栓发生率，与以往研究结果一致，且发生血栓患者的TPO-RA使用率高于未发生血栓患者。

ITP患者的高复发率同样是目前治疗中亟待解决的问题之一，一项研究表明，接受标准一线治疗的ITP患者约50％在治疗后6个月内复发[25]。另一项中国研究统计了233例成人ITP患者的复发情况，其中85例患者在6个月内复发，复发率为36.4％[26]。关于ITP复发的相关危险因素，有研究指出在儿童中前驱感染、治疗前病程、初次治疗血小板升至有效止血时间属于独立危险因素[27]。在成人中复发则与治疗前平均血小板体积（MPV）和血小板淋巴细胞比值（PLR）相关，其中MPV低于21 fl是ITP复发的独立危险因素[26]，而PLR处于一定范围内时其增加与复发减少相关[28]，但复发的相关危险因素还需进行更多的研究。

ITP的症状和体征以及治疗相关不良反应（自发性瘀伤、月经过多、黏膜出血等）可能会显著影响ITP患者的生活质量（HRQoL）有必要研发并选择合适的量表对ITP患者的HRQoL进行评估。对于成人ITP患者，目前国内外常用的工具有SF-36量表、慢性病治疗疲劳功能评估（FACIT-F）、ITP-PAQ等，对于儿童则主要使用PedsQL、KIT两种工具。其中，SF-36及FACIT-F为非特异性量表，其无法获得有关ITP特定QoL问题的信息，例如患者的出血症状及对出血的恐惧心理等，而ITP-PAQ则是2007年由Mathias等编制的的特异性评估成人ITP的综合HRQoL问卷，该量表的多种语言版本已在不同国家的ITP患者中得到验证，具有良好的可靠性和有效性[4],[29]–[30]。本研究使用的调查问卷即以ITP-PAQ为基础，在收集问卷后使用李克特量表计分法将每一项的结果转换为分数并进行分析。

目前，有多项研究使用多项研究证实，与对照组或一般人群相比，ITP患者的生活质量降低，特别是在身体和社交活动方面[31]–[33]。疲劳是ITP患者中公认的最常见症状之一，且持续性ITP患者的疲劳负担明显高于新诊断/慢性患者[32],[34]–[35]。本研究调查结果显示，ITP患者的QoL在生活质量、困扰、运动、心理、恐惧几个维度的得分均较低，一半以上患者报告了疲劳/乏力相关症状，且患者的疲劳/睡眠、困扰、心理、生活质量维度的评分与患者的出血症状情况无关（|r|<0.3）。ITP患者心理状态及情绪易受疾病症状及治疗的影响，大部分ITP患者对出血症状、感染及死亡有不同程度的担忧。另外，在本研究中，ITP患者在各维度的得分与其性别和年龄无关（*P*>0.05），与以往研究保持一致[36]。

在早期临床指南和实践中，国内外医生对ITP患者的生活质量重视不足，医生习惯于通过血小板计数及患者出血症状来评估疗效。回顾2010至2019年国内外发表的168项关于ITP患者治疗反应的研究，只有15项（9％）评估了ITP患者的HRQoL结果[37]。另外此前在国际大型研究I-WISH的中国亚组研究中也显示，中国医生在患者随访时往往更多关注紫癜和月经过多等躯体症状，而对ITP患者疲劳和焦虑症状认识不足[38]。随着医生及患者对ITP认识的增加，ITP患者的生活质量管理逐渐成为治疗的重要组成部分，改善ITP患者生活质量已经被视为ITP三大治疗目标之一。ITP患者生活质量管理需要医患双方的共同努力。对患者来说，可以通过自我监测、放松训练、保持健康的生活方式、主动寻求帮助等方式应对疾病带来的不良情绪；从医生角度来说，在确定治疗方案时，医生需要综合考虑患者的身体状况、心理状态和社会支持，鼓励患者参与治疗选择，在治疗不良反应最小化基础上提升血小板计数至安全水平，减少出血事件，从而达到最佳的治疗效果。同时，医疗机构可以通过定期随访、举办患教活动等方式及时了解患者的治疗及恢复情况，提高患者对ITP疾病的认识，从而缓解患者对疾病的担忧和焦虑情绪。近年来，已有越来越多的研究者将ITP患者生活质量评分纳入ITP治疗的观察指标和疗效评价中，随着对ITP临床研究的深入和对ITP患者生活质量的重视，ITP的治疗及管理将更加规范化、个体化。

本研究有一些局限性：①在评估ITP患者的出血症状时，仅统计了不同类型出血症状的比例，未对出血的严重程度进行客观性评分；②受数据收集的限制本研究未能对ITP患者临床特征和疗效、预后、不良反应的相关性开展更深层次的分析；③本研究进行生活质量分析时，发放的问卷回收率较低，导致纳入研究的样本数量较小，且受线上填写方式的影响，可能导致填写问卷的老年人/儿童比例偏低，因此应谨慎解释本研究的结果，未来还需要更多大规模、多中心的数据对ITP患者的生活质量和相关影响因素进行调查和分析。

综上，本研究回顾了湖北省七家医疗中心ITP患者的诊治情况及ITP对患者生活质量的影响，总结并讨论了目前ITP的诊断、治疗进展，生活质量情况。ITP疾病慢性化比例较高，糖皮质激素、促血小板生成药物在ITP患者的治疗中占据重要地位。在治疗上应坚持个体化原则，尽量选择成本效益更高的治疗方案，重视ITP患者生活质量管理，将恢复和（或）维持ITP患者的生活质量视为治疗的重要目标。
